# Host Manipulation, Gene Editing, and Non-Traditional Model Organisms: A New Frontier for Behavioral Research?

**DOI:** 10.3389/finsc.2022.938644

**Published:** 2022-07-07

**Authors:** Jean-François Doherty, Benjamin J. Matthews

**Affiliations:** Department of Zoology, University of British Columbia, Vancouver, BC, Canada

**Keywords:** host manipulation, gene editing, non-traditional model organism, CRISPR-Cas9, insect, behavior

## Abstract

Insects and parasites dominate the biosphere, in terms of known biodiversity and mode of life, respectively. Consequently, insects play a part in many host-parasite systems, either as parasite, host, or both. Moreover, a lot of these systems involve adaptive parasite-induced changes of host phenotype (typically behavior or morphology), which is commonly known as host manipulation. While many host manipulation systems have been described within the last few decades, the proximate mechanisms that underpin host phenotypic change are still largely unknown. Given the intimate co-evolutionary history of host-parasite systems, teasing apart the intricate network of biochemical reactions involved in host manipulation requires the integration of various complementary technologies. In this perspective, we stress the importance of multidisciplinary research on host manipulation, such as high-throughput sequencing methods (genomics and transcriptomics) to search for candidate mechanisms that are activated during a manipulation event. Then, we argue that gene editing technologies, specifically the CRISPR-Cas9 system, are a powerful way to test for the functional roles of candidate mechanisms, in both the parasite and the host. Finally, given the sheer diversity of unique host-parasite systems discovered to date, there is indeed a tremendous potential to create novel non-traditional model systems that could greatly expand our capacity to test the fundamental aspects of behavior and behavioral regulation.

## Introduction

Insects have dominated Earth’s animal biodiversity for hundreds of millions of years (1–3). Parasitism, wherein one organism typically exerts a negative fitness effect upon a host organism by living in or on it, has arguably become the dominant mode of life on this planet (4, 5). Therefore, unsurprisingly, most lineages within Metazoa that have independently evolved parasitism consist of insects and other arthropods (6). In return, insects play host to a vast range of parasites, including viruses (7), protists (8), fungi (9), helminths (10), and other insects (11). Since it is likely that every metazoan species has evolved with at least one parasitic species (12), and most animal species, i.e., insects, remain to be discovered and formally described (13, 14), there is undoubtedly an enormous and currently underappreciated diversity of unique host-parasite systems (5, 15). Moreover, each of these systems reflect an entire co-evolutionary history that has resulted in a number of adaptations for both antagonistic species, creating a complex network of host-parasite interactions spanning multiple biological systems. While parasites are typically adapted to avoid or resist the host defense system in order to develop (16), hosts are usually adapted to avoid or defend against parasitic infection (17, 18). However, many parasitic lineages have evolved other remarkable strategies that ultimately tip the odds of success in their favor.

A parasite that adaptively changes the phenotype of its host to increase the likelihood of completing its life cycle is said to “manipulate” its host. While this metaphor promotes a parasite-centric perspective of the phenomenon (19), host manipulation has been highlighted as one of the most influential ideas in parasitology (20). Since adaptive host manipulation was used to explain the behavioral changes observed in amphipod hosts during the early 1970s (21), hundreds of host-parasite systems requiring host manipulation have been described (22, 23). Many of these include insects as either the parasite, the host, or both, and some of the most notorious examples of host manipulation involve the altered behaviors of insect hosts (24): hairworms (phylum Nematomorpha) cause their terrestrial insect hosts to enter water, where the host typically dies and the hairworm escapes to mate (25); lancet liver flukes (phylum Platyhelminthes) cause their intermediate ant hosts to climb to the tip of grass blades to be eaten by grazing ruminants, allowing the parasites to pursue their development (26); and jewel wasps (Hymenoptera: Ampulicidae) render their cockroach hosts nearly incapable of walking voluntarily, allowing the wasp to bring the host back to its nest and lay its eggs upon it (27). Throughout the past few decades, many researchers have helped catalogue new host manipulation systems, and in recent years have been working to identify the proximate mechanisms and molecular interactions at play (20). If a parasite is truly capable of adaptively manipulating its host, we should be able to (i) pinpoint the “manipulation gene(s)” encoded in the parasite genome and (ii) trace the causal chain between these gene(s) and the phenotypic changes observed in the host (24, 28–30).

Within the past decade or so, there have been growing calls for multidisciplinary research on host-parasite interactions to uncover the proximate nature and true extent of host manipulation by parasites (29–33). Given their sheer diversity, insects play an important role as parasite or host in many host manipulation systems, representing a key source of potential model systems for host manipulation research (24, 34). In this perspective, we highlight recent integrative and multidisciplinary research on insects that has shed light on some of the complex interactions occurring during host manipulation. Then, we call attention to a recent development in gene editing technology (CRISPR-Cas9) as a powerful tool to test the causal roles for genetic loci, in both host and parasite, that modulate host phenotype. We argue that these state-of-the-art technologies, in combination with host manipulation, will lead to the creation of novel, non-traditional model systems that will expand upon the behavioral studies of traditional, laboratory models. Finally, we conclude that host manipulation systems, involving two closely interacting species that have adapted fine-tuned mechanisms in an intimate evolutionary arms race, offer researchers a unique and powerful perspective on understanding the fundamental nature of behavior and behavioral regulation.

## Multidisciplinary Research on Host Manipulation

**Figure 1:**
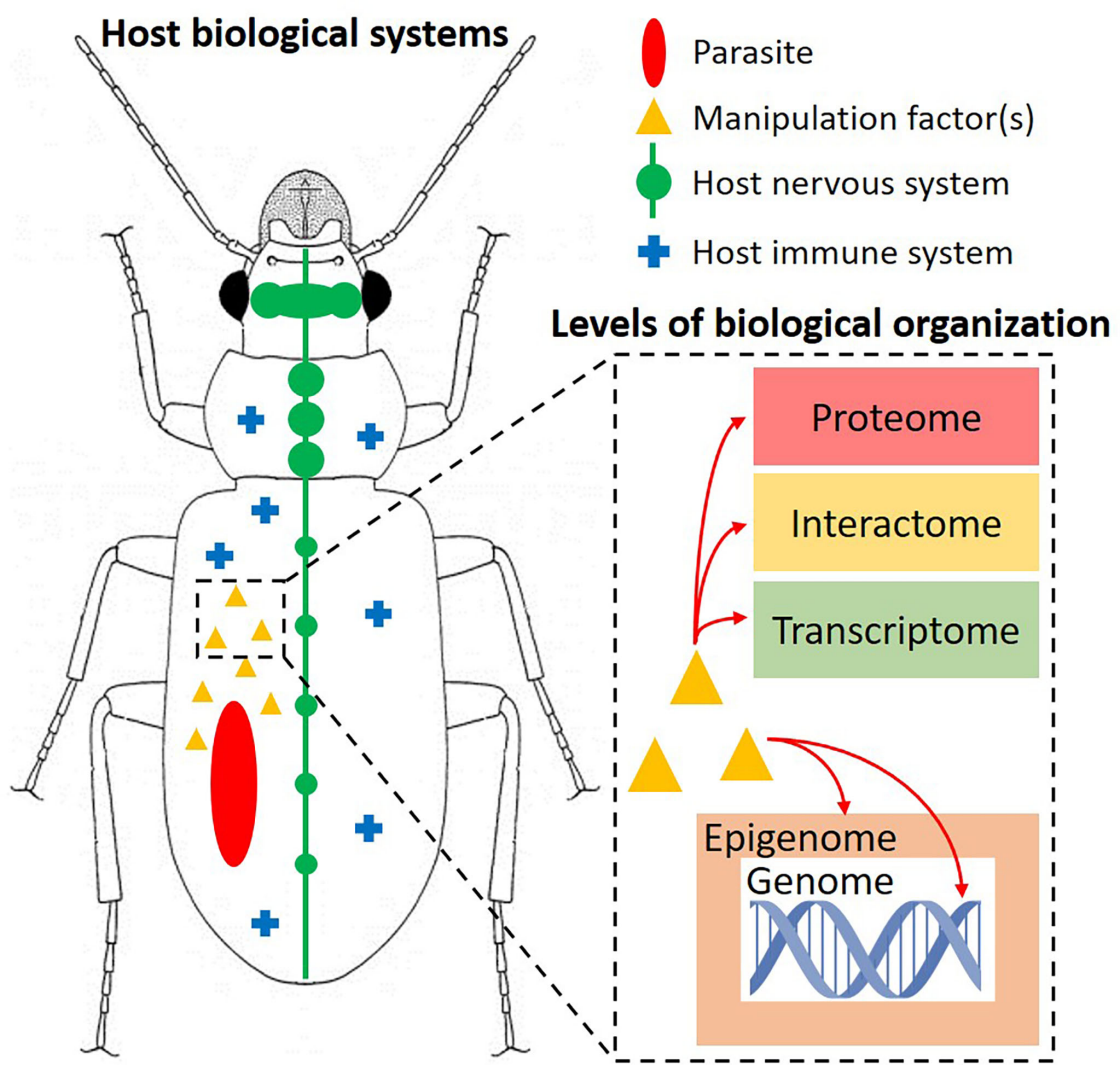
Depicting the multidimensional nature of host manipulation in insects. A parasite produces “manipulation factor(s)” that can interact with and cause cascading effects in various host biological systems, such as the nervous and immune systems. These manipulation factor(s) can impact the host at one or more levels of biological organization (dashed rectangle), from the genome to the proteome. These interactions ultimately result in adaptive changes in host external phenotype (typically behavior or morphology) that benefit the parasite. Note that the parasite was haphazardly placed inside the insect; its location varies with each system and can play an important role during manipulation. Figure inspired by Hébert and Aubin-Horth (37) and Herbison (38).

The adaptations initially required for parasites to evade or resist host immunity could have resulted in fortuitous, indirect mechanisms that were then honed by natural selection to produce novel, directed mechanisms necessary to alter host phenotype and favor parasite development (35, 36). Given their indirect origins, these adaptations could have variable impacts on multiple host systems (**Figure 1**), highlighting the multidimensionality of host manipulation (39). To complicate things even more, these impacts may affect the host at multiple levels of biological organization, from the epigenome (epigenetic markers) to the protein interactome (network of host-parasite protein interactions) (33, 37) (**Figure 1**). Therefore, uncovering the proximate mechanisms of host manipulation naturally requires an in-depth, multidisciplinary approach, integrating experimental infections, various “-omics”, specialized imaging technologies, etc. (24). For example, proteomics was used relatively early in host manipulation research to identify differences in protein expression profiles between infected and uninfected individuals of various host-parasite systems (mainly insect hosts), with the goal of identifying candidate gene function during a host manipulation event (40–42). Other studies have utilized advanced imaging technologies to locate parasites in their insect hosts, thus recognizing the importance of the parasite’s physical presence and exact location during host manipulation (43, 44). Identifying candidate mechanisms is one thing, but more importantly is the ability to control or suppress these mechanisms in order to test for causation, rather than just observing a correlation of events during manipulation. While the importance of integrating different approaches in host manipulation research has been brought forward in recent reviews (24, 38), only within the last few years have we seen studies that have exploited different technologies to explore the underpinnings of host manipulation.

High-throughput sequencing technologies have been described as the next best thing in host manipulation research (24, 32, 37, 45), and rightly so. The ability to quantify the complete set of RNA transcripts (transcriptomics) at a precise moment in time or at a specific location in the organism (host or parasite) can allow us to identify the genes that are over- or under-expressed during a manipulation event. In turn, comparative genomics and transcriptomics, i.e., comparing the sequences or expression levels of genes between species, have opened the door to identifying signatures of convergent evolution, protein mimicry, or other shared mechanisms between host and parasite that might be responsible for altering host phenotype. Despite the potential power of these techniques, only a few studies on host manipulation have utilized transcriptomics to identify candidate genes or mimicry proteins between host and parasite (46–48). For example, Will et al. (48) used comparative transcriptomics before, during, and after manipulation to uncover a network of candidate genes that were linked to host circadian rhythm, foraging behaviors, neuromodulation, and novel parasite compounds and toxins - all biological pathways thought to be important for the successful manipulation of ants by *Ophiocordyceps* fungi. In this well-documented system, infected ants climb up vegetation, clamp down on the underside of leaves with their mandibles, and die, where the fungus can produce a fruiting body and spread its spores by shedding them upon other ants (49). Undoubtedly, this type of integrative research can help uncover the candidate mechanisms involved in host manipulation events, but linking these mechanisms to functional and observable changes in host phenotype will require a completely different type of technology, one that harnesses the remarkable properties of a certain prokaryotic immune system.

## CRISPR-Cas9 and Host Manipulation

At the time when researchers were beginning to explore the adaptive nature of host manipulation by parasites (21), others were starting to understand the role of certain prokaryotic molecules in bacterial immunity and their potential for manipulating DNA (50–52). Through the following decades, more and more prokaryotic enzymes capable of binding to specific DNA sequences and breaking the double-stranded helix were discovered (see 53 for a brief history). Among these, the *Streptococcus pyogenes* CRISPR-Cas9 system was recognized for its robustness, simplicity, and flexibility and, with the understanding that living cells can incorporate exogenous DNA through homologous recombination (54) and other homology-directed repair, provided the promise of a true “cut-and-paste” genome engineering tool. CRISPR-Cas9 eventually replaced older technologies, including zinc-finger nucleases and TALENs, as the primary technology used in targeted gene editing, due to its ease of reprogramming (53, 55, 56). Essentially, a CRISPR (clustered regularly interspaced short palindromic repeat) is a prokaryotic DNA sequence consisting of short palindromic repeats and non-repeating spacers (57). Spacers are DNA sequences naturally acquired from attacking bacteriophages and integrated into the bacterial genome (58, 59). Found in most Bacteria and Archaea (60), these spacers are transcribed into short CRISPR RNAs (crRNAs), which guide associated Cas (CRISPR-associated) enzymes to target and cut a specific region of the bacteriophage genome and defend against invading nucleic acids (61). Researchers were able to reprogram the *S. pyogenes* Cas9 enzyme to target specific DNA sequences in bacteria by generating short guiding RNA sequences *in vitro* (62) and, in just a few years’ time, the CRISPR-Cas9 system was successfully adapted for *in vivo* genome editing in eukaryotic cells (63–65). Within the past decade, researchers from many fields have adopted CRISPR-Cas9 technologies, as well as identifying other CRISPR-Cas systems, which have become the method of choice for genome engineering (53, 55, 56).

The latest developments of CRISPR gene editing technologies have greatly expanded upon the original CRISPR-Cas9 system, such as CRISPR activation (CRISPRa) and CRISPR interference (CRISPRi), which essentially up- or down-regulate gene expression, respectively (56). Unsurprisingly, CRISPR-Cas9 gene editing is now being used to uncover the molecular determinants of pathogenesis for a number of important human diseases such as dengue fever and malaria (66, 67). Increasingly, this system is being adapted to a variety of parasites, including bacteria, protists, and nematodes, to study intricate host-parasite interactions and uncover functional traits encoded in parasite genomes (68–70). Although these studies have focused on disease-causing agents in humans, there is indeed a great potential to combine CRISPR-Cas9 with other areas of parasitological research, such as host manipulation. Previously, we highlighted the capability of identifying candidate parasite genes and shared mechanisms between host and parasite through comparative genomics and transcriptomics. While this is surely a crucial step in discovering the molecular changes responsible for host phenotypic change, the data obtained from comparative studies are still correlational in nature. Since parasites can impact host phenotypes across multiple systems (especially the immune and nervous systems) and levels of biological organization (36, 37) (**Figure 1**), there is bound to be a large amount of molecular noise associated with any biochemical reactions occurring simultaneously or coincidentally in the host (or parasite) during the manipulation period. Even so, if the “manipulation factors” of a parasite have a large number of cascading effects in the host (30), natural selection has likely optimized the specific pathways that ultimately lead to an increase in parasite transmission (28).

**Figure 2:**
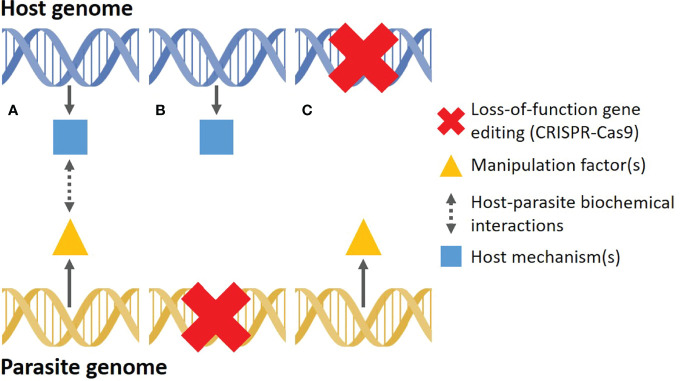
Simplified depiction of how loss-of-function CRISPR-Cas9 gene editing can be used to elucidate the proximate mechanisms of host manipulation. **(A)** During a natural manipulation event, “manipulation factor(s)”, encoded in the parasite genome, are released into the host. These factors initiate a series of host-parasite interactions that impact one or more host mechanisms encoded in the host genome, resulting in external changes in host phenotype (typically behavior or morphology). **(B)** CRISPR-Cas9 is used on a living parasite to knockout a candidate parasite gene with potential manipulation functions, allowing researchers to test which parasite genes have a functional role during manipulation. **(C)** CRISPR-Cas9 is used on a living host to knockout a candidate gene potentially manipulated by the parasite, allowing researchers to test which host mechanisms underlie the altered phenotype.

The ability to remove specific genes (gene knockout) in any genome using CRISPR-Cas9 will essentially allow us to test the causative and functional role of candidate mechanisms identified during a natural manipulation event (24) (**Figure 2A**). Knocking out candidate genes in the parasite would allow us to test for any direct causal link between the knockout gene and host phenotype (**Figure 2B**), whereas knocking out genes in the host would help us determine what host mechanisms are directly impacted by parasite manipulation (**Figure 2C**). To help illustrate this concept, we look at the discovery of the mechanism responsible for tree-top disease in moth larvae infected with a baculovirus. In this relatively simple system, infected larvae are prone to climb to the top of trees to die, where they liquefy and release viral particles, thus infecting other larvae below (71). Early on, it was shown that ecdysteroid UDP-glucosyltransferase, encoded by the *egt* gene in the virus, prevents larvae from molting by inactivating the larval molting hormone 20-hydroxyecdysone (72). The *egt* gene was also hypothesized to play a role in the tree-climbing behavior of infected larvae. Recombinant viruses, lacking the *egt* gene, were inoculated in larvae and the climbing behavior almost completely disappeared in comparison to larvae inoculated with wild-type viruses (73). In a closely related system, CRISPR-Cas9 was recently used to knockout three candidate host genes, all important for host visual perception pathways (74). These genes were found, through transcriptomics, to be upregulated after viral infection, causing an acute phototactic response and increased climbing in larvae. Infected knockout larvae showed a significantly reduced climbing behavior, strongly implying that these specific host genes are important for the successful manipulation by the baculovirus (74). To truly confirm which gene(s) underpin the manipulation of larvae, CRISPRa could be used to activate specific candidate host genes to try and replicate the climbing behavior observed in naturally infected individuals. Albeit simple on paper, this is a nascent field of research and its scope is currently very limited. In fact, the logistics of working with a new organism in a laboratory setting is a prerequisite for genetic editing approaches and can often be much more challenging than designing and generating the necessary CRISPR-Cas components (75). Parasites may pose additional challenges as they require a host to develop, which can complicate their maintenance and manipulation under artificial conditions. This would make CRISPR-Cas9 in parasites particularly challenging, although novel techniques, such as ReMOT Control (76) and DIPA-CRISPR (77), may increase the success rates of knockouts (or knock-ins) in these systems. Despite these challenges, gene editing promises to provide us with a powerful and elegant method to test the functional roles of host-parasite mechanisms involved during host manipulation.

## A Plethora of Non-Traditional Model Organisms for Behavioral Research

In the previous sections, we featured the recent developments of multidisciplinary research on host manipulation in insects, and highlighted the potential of CRISPR-Cas9 gene editing as an effective method to elucidate the functional mechanisms causing host behavioral change. This type of research stresses the importance of comparative studies and the strength of integrative research on behavior (33, 78). Host-parasite systems arguably represent an untapped source of potential research on behavioral regulation and the genes that govern it. Traditional model systems can allow for decades-long research and in-depth investigations into behavior (79). However, the molecular pathways under scrutiny in these systems are limited to the evolutionary history of the model organism. Contrastingly, non-traditional model organisms can offer us a better glimpse of the entire spectrum of biological diversity, greatly expanding our understanding of the adaptations and molecular mechanisms underpinning animal life on this planet (80). Researchers have even begun outlining ways to create novel model systems, integrating high-throughput sequencing and genome editing, in the goal of branching out from the widely used traditional models (75). Given the known diversity of host-parasite systems involving insects and host manipulation, and the potential of discovering many more of these systems, it is time to start harnessing the power of genome engineering to explore the fine-tuned mechanisms that parasites have acquired through natural selection to manipulate their hosts.

## Concluding Remarks

In this perspective, we have featured some of the vast diversity of host-parasite interactions that occur in insects. Many of these systems require parasite-induced host phenotypic changes (mainly behavior or morphology) that benefit the parasite, a phenomenon commonly known as host manipulation. Given the evolutionary history of host-parasite interactions and the sheer diversity of insects (14, 36), many examples of host manipulation involve behavioral alterations of insect hosts (22). Parasites can impact host behavior through multiple systems and levels of biological organization. Therefore, multidisciplinary research is a necessary step towards elucidating the fundamental nature of host manipulation and behavioral regulation. We showed that comparative genomics and transcriptomics can help identify candidate genes and mechanisms that are activated during manipulation. While this is a crucial step in uncovering the molecular underpinnings of host manipulation, these data remain correlational in nature. To truly test the functional roles of these candidate mechanisms, gene editing technologies offer us a powerful means of assigning causation to the genes responsible for the host phenotypic alterations under scrutiny. In this regard, the CRISPR-Cas9 system has revolutionized the way in which researchers can target and modify specific areas of the genome in living organisms (53). Finally, we argued that host-parasite interactions represent a huge untapped source of non-traditional study systems that, paired with multidisciplinary research, could greatly broaden the horizons of ethology. While parasitologists have occasionally been slow to adopt novel technologies and molecular methods for their research (81), host manipulation, gene editing, and integrative research could pave the way for fundamental studies on behavioral regulation.

## Data Availability Statement

The original contributions presented in the study are included in the article. Further inquiries can be directed to the corresponding author.

## Author Contributions

J-FD conceptualized the idea and wrote the manuscript. BM provided critical input throughout. All authors contributed to the article and approved the submitted version.

## Conflict of Interest

The authors declare that the research was conducted in the absence of any commercial or financial relationships that could be construed as a potential conflict of interest.

## Publisher’s Note

All claims expressed in this article are solely those of the authors and do not necessarily represent those of their affiliated organizations, or those of the publisher, the editors and the reviewers. Any product that may be evaluated in this article, or claim that may be made by its manufacturer, is not guaranteed or endorsed by the publisher.
